# Supplementary material for: Ceftazidime resistance in Pseudomonas aeruginosa is multigenic and complex
Source: PLoS One. 2023 May 16;18(5):e0285856. doi: 10.1371/journal.pone.0285856 (PMC10187892; doi:10.1371/journal.pone.0285856)
Supplement: Attachment — Submitted filename: PONE-D-23-06436_Response to reviewers.docx [file pone.0285856.s002.docx]

Dear Dr Omri

**Response to reviewers’ comments on manuscript PONE-D-23-06436**

Thank you for providing the reviewers’ comments on our submitted manuscript. We are grateful to the reviewers for the time spent in considering our manuscript, and for their constructive suggestions on how to improve it. We have now adjusted the manuscript in light of the reviewers’ comments and to clarify issues that they have raised.

A point-by-point response to all of the reviewers’ comments on the earlier version of this manuscript is given below. Reviewers’ comments are shown in normal font, with our responses in italics. All references to line numbers in our responses refer to the numbering in the marked up revised manuscript.

We hope that our manuscript will now be acceptable for publication in PLoS One.

We will look forward to hearing from you.

Kay A Ramsay, Corresponding author on behalf of all the authors.

**Reviewer #1**: This manuscript by Ramsay and colleagues investigates how genetic mutations interact to confer resistance to ceftazidime. It is clearly written and logically presented; the experiments are technically sound. A few comments for the authors' consideration:

1. Is there enough information in the data presented in Table S2 and 3 to understand something about the order in which mutations might occur? Are there mutations that only occur conditioned on the acquisition of another mutation? For instance, it appears that in PA14 clpA mutations only occur if there is also a phoQ mutation, but not the other way around. This kind of analysis, if it is possible, would add to the robustness of the discussion.

*The referee raises an interesting question. Unfortunately it is not possible from our data to determine the order in which mutations occur. Although clpA mutants were only present in bacteria with phoQ mutations, there is not enough information to determine whether this relationship is meaningful, given that the majority of the PA14 mutants have phoQ mutations.*

2. Can the authors please discuss why there is almost no (sometimes a negative) change in meropenem resistance in the engineered PAO1 variants?

*Resistance to meropenem arises primarily through mutations altering the OprD protein that allows access of meropenem into the bacterial cells, and to the PBP3 protein that is the target for meropenem. Mutations in these genes did not arise in our study indicating that mechanisms of resistance to ceftazidime and meropenem are different. The engineered mutants had mutations in genes associated with ceftazidime resistance but not meropenem resistance explaining why the mutants did not have large increases in meropenem resistance – for example, mutations in dacB do not increase the MIC for meropenem. An exception is the nalD mutation in which mutations cause upregulation of an efflux pump that causes small increases the MIC for both ceftazidime and meropenem.*

*Although there is an apparent small decrease in meropenem MIC in two of the engineered mutants (Table S5), the MIC replicates show that the PAO1 MIC was only marginally above these and the difference is very minor.*

3. It's not at all clear what the transposon mutant table (Table 4) adds to the story. The authors have already gone through the trouble of making a variety of deletions and point mutants!

*The transposon mutants were used essentially to independently confirm the results with the engineered mutants. In addition however, as insertions, they represent complete gene knockouts in contrast to some of the point mutations that may only partially remove protein function, or may be gain-of-function mutations.*

**Reviewer #2**: Useful study analyzing the effect of individual and combined mechanisms leading to ceftazidme resistance in P. aeruginosa obtained after in vitro evolution experiments using PAO1 and PA14 strains. There are however some points for the authors to consider:

1. Lines 48-49. There are other well established mechanisms leading to AmpC overexpression such as inactivation of AmpD or specif AmpR mutations.

*We have now included reference to AmpD and AmpR in the introduction (lines 47-50 in the revised manuscript).*

2. Related to this, it is somewhat surprising that none of the evolved mutants showed AmpD mutations. It should be commented in the discussion.

*Mutations in ampD have been associated with ceftazidime resistance in two previous experimental evolution studies, though are not invariably present in resistant mutants. One of the mutants in our study had an ampD mutation. It is not clear why the frequency of ampD mutations was not higher; we speculate that this may due to differences in the selection protocols used (e.g agar plates versus broth culture) and have now included this suggestion in the revised manuscript (lines 299-302).*

3. Table 5. Difficult to interpret without expression data for ampC and mexB. A limitation statement should be added.

*The purpose of this section of the paper was to determine the frequencies of mutations in genes of interest in ceftazidime-sensitive and -resistant isolates, rather than to determine the mechanisms of resistance. We agree with the reviewer that the contribution of variants to resistance will be affected by the level of gene expression and we have expanded the text to make this clear for ampC (lines 259-260). As the numbers of variants in mexB were relatively low and not very different between sensitive and resistant isolates, we have not specifically mentioned mexB in this context.*

4. Lines 287-289. PAO1 dacB mutant is the only one showing clinical resistance levels

*As implied by the reviewer, the dacB mutation did not by itself confer clinical resistance levels and we have rephrased and expanded the text (lines 290-291) to make this clearer.*

5. Lines 293-294 increase in ampC expression produced by mpl inactivation much lower than that produced by dacB or ampD inactivation.

*We agree that this is a possible explanation for the different effects of mpl and dacB mutations in our study – we did not analyse ampD. However we did not measure the effects of mutations on gene expression, and we are not aware of studies comparing the effects of mpl, dacB and ampD mutations on expression, and so discussion of this would be rather speculative.*

6. Line 295-298. Usually AmpC mutations require AmpC overexpression to have a significant effect and therefore expected to add synergistic with dacB mutations. Frequently these AmpC mutations decrease MIC for carbapenems.

*The ampC dacB double mutants had higher MICs than either ampC or dacB single mutants (Table 3), consistent with the reviewer’s comment. Two ampC mutations were examined for their effects with a single carbapenem (meropenem) and neither caused a striking decrease in MIC. It is likely that mutations other than those studied here can cause the collateral sensitivity noted by the reviewer.*

7. Lines 336-345. Usually these deletions show other typical characteristic such as brown pigment (hmgA deletion) and colistin (galU deletion) and aminoglycoside (meXY deletion) hypersusceptibility.

*We agree that this is the case, and several of the deletion-containing mutants in this study had brown pigment. We did not test whether the other phenotypes were altered in deletion-containing mutants. The mutants contained multiple other mutations that may also affect colistin or aminoglycoside susceptibility and so changes to these phenotypes would not be clearly resulting from the deletions.*

8. Some other expected mechanisms such as PBP3 mutations were not detected and might be discussed.

*We have considered this suggestion. Mutations in the ftsI gene that encodes PBP3 contribute to resistance to several b-lactams but their contribution to ceftazidime resistance is less clear. To the best of our knowledge, in previous studies experimental evolving ceftazidime-resistant mutants only one mutant has had an ftsI mutation. It is therefore not easy to know what to make of their absence in this study. Rather than expanding the introduction and discussion we consider it best to omit specific reference to ftsI. With regard to other mechanisms, we have incorporated reference to ampD as described above.*

9. For discussion. A recent work determined a genomic score for predicting ceftazidime (and other antibiotics) resistance (Cortés-Lara et al CMI 2021).

*We thank the reviewer for bringing this paper to our attention, we have now incorporated it into the manuscript (Introduction and Discussion) and added a sentence to the discussion (lines 375-378) on the important topic of predicting antibiotic resistance from genome sequence.*
